# Genome-wide association studies and traditional Chinese medicine in hyperuricemia: current evidence and mechanistic insights

**DOI:** 10.3389/fphar.2025.1647264

**Published:** 2026-01-12

**Authors:** Le Yang, Jing Liu, Shengping Luo, Yihui Deng

**Affiliations:** 1 School of Integrated Chinese and Western Medicine, Hunan University of Chinese Medicine, Changsha, China; 2 Hunan Province Key Laboratory of Cerebrovascular Disease Prevention and Treatment of Integrated Traditional Chinese and Western Medicine, Hunan University of Chinese Medicine, Changsha, China; 3 School of Traditional Chinese Medicine, Hunan University of Chinese Medicine, Changsha, China

**Keywords:** Chinese medicine, genome-wide association study, hyperuricemia, urate transporters, uric acid homeostasis

## Abstract

Hyperuricemia is a significant risk factor for gout, cardiovascular disease, and chronic kidney disease, and its global prevalence has continued to rise. Genome-wide association studies (GWAS) have made significant advances in elucidating the genetic basis of serum uric acid levels, identifying key loci such as *SLC2A9*, *ABCG2*, *SLC22A12*, *GCKR*, and *HNF4A*, while also revealing population heterogeneity and gene–environment interactions. Concurrently, traditional Chinese medicine (TCM) has demonstrated multi-component, multi-pathway regulatory effects on uric acid production, renal and intestinal excretion, inflammatory responses, and gut microbiota. This review summarizes recent GWAS advances in hyperuricemia and compiles experimental and mechanistic studies on TCM regulation of uric acid homeostasis over the past 5 years. Furthermore, the discussion section outlines current limitations in both GWAS and TCM research, proposes potential connections between them in specific regulatory processes, and explores possible directions for future mechanistic studies and the development of intervention strategies.

## 1 Introduction

Hyperuricemia is a metabolic disorder caused by abnormal purine metabolism or impaired uric acid excretion. According to the *China multi-disciplinary expert consensus on diagnosis and treatment of hyperuricemia and related diseases (2023 Edition)*, hyperuricemia is diagnosed when fasting serum uric acid levels exceed 420 μmol/L (approximately 7.0 mg/dL) on two separate occasions (Fang et al., 2023). With shifts in lifestyle and dietary patterns, the global prevalence of hyperuricemia continues to rise (Chen-Xu et al., 2019; Zhang M. et al., 2021). Beyond gout attacks, hyperuricemia is closely associated with hypertension, cardiovascular disease, metabolic syndrome, and chronic kidney disease (Saito et al., 2021; Keenan, 2020), posing a significant public health challenge.

Uric acid primarily originates from endogenous purine metabolism, with approximately 70% excreted via the kidneys and the gut serving as the main auxiliary pathway (Keenan, 2020). Hyperuricemia may result from increased uric acid production or impaired renal/intestinal excretion. Commonly used urate-lowering drugs include allopurinol and febuxostat, which inhibit uric acid synthesis, and benzbromarone, which promotes excretion. However, these drugs carry adverse effects such as hepatotoxicity, nephrotoxicity, and severe hypersensitivity reactions (Ishikawa et al., 2020). Necessitating the exploration of new targets and safer, more effective therapeutic approaches.

In recent years, genome-wide association studies (GWAS) have significantly advanced the genetics of hyperuricemia, identifying a series of key genes influencing serum uric acid levels and providing crucial insights into genetic susceptibility and regulatory pathways. Concurrently, Traditional Chinese Medicine (TCM) has accumulated extensive experience in reducing uric acid, improving renal function, and regulating metabolism. Its multi-component, multi-target, and multi-pathway mode of action continues to attract sustained attention. However, there remains a lack of reviews that integrate hyperuricemia research progress from dual perspectives: genetic regulation and TCM mechanisms of action.

Against this backdrop, this paper compiles major GWAS findings on hyperuricemia, summarizes advances in TCM research on regulating uric acid homeostasis over the past 5 years, and discusses potential intersections between genetic evidence and TCM mechanisms, with the aim of informing future target discovery and the optimization of intervention strategies.

In this review, all botanical names mentioned in Traditional Chinese Medicine (TCM) studies were taxonomically verified using authoritative databases, including the Medicinal Plant Names Services (MPNS) and Plants of the World Online (POWO), to ensure the accuracy and consistency of species nomenclature.

## 2 Literature search strategy

This study employed a structured literature search approach, developing distinct strategies for the two themes: “GWAS studies on hyperuricemia” and “Mechanisms of uric acid homeostasis regulation by TCM.”

For GWAS studies on hyperuricemia, we searched the PubMed database (including the GWAS Catalog) from 2007 to 2025. The year 2007 was selected as the starting point because it marked the first publication of a GWAS study demonstrating a significant association between *SLC2A9* and serum uric acid (Li et al., 2007), signifying the inception of uric acid genetics research. Search terms included “hyperuricemia,” “serum urate,” “genome-wide association study,” and “GWAS.” Inclusion criteria were: 1. Serum uric acid or hyperuricemia as the primary phenotype; 2. Use of GWAS or related statistical genetic methods; 3. Peer-reviewed original research or review articles. The screening process involved initial title/abstract screening and full-text review, ultimately yielding approximately 100 included studies.

For mechanism studies of TCM in hyperuricemia, a combined search of PubMed, Web of Science, and Scopus databases was conducted using terms including “hyperuricemia,” “traditional Chinese medicine,” “mechanism,” and “herbal medicine.” The search period was restricted to 2020–2025 to reflect the recent surge in mechanism studies. Inclusion criteria were: 1. Hyperuricemia as the explicit research subject; 2. TCM formulas limited to classical prescriptions; 3. Single herbs and active components must be listed in classical Chinese pharmacopoeias or modern pharmacopoeias; 4. Peer-reviewed experimental studies, clinical trials, or systematic reviews. Exclusions included small-sample case reports, non-peer-reviewed literature, single-intervention Western medicine studies, and duplicate reports. Approximately 80 studies were ultimately included.

All literature was independently screened by two researchers, with disagreements resolved through discussion to ensure rigorous and reproducible retrieval processes.

## 3 GWAS discoveries in hyperuricemia

Current GWAS research on hyperuricemia primarily focuses on three areas: First, systematically identifying core genetic loci influencing serum uric acid levels and elucidating their functions in key pathways such as purine metabolism, renal tubular reabsorption, and intestinal excretion. Second, assessing genetic effect heterogeneity across different populations and genders to reveal the role of genetic structural differences in disease susceptibility. Third, investigate the interactions between genetic factors and environmental exposures, such as diet, alcohol, medications, and lifestyle factors, to elucidate how genes and environmental factors jointly shape hyperuricemia risk. These studies not only deepen our understanding of the genetic regulation of uric acid homeostasis but also provide crucial clues for subsequent target validation and personalized interventions.

### 3.1 Core susceptibility genes

GWAS studies of hyperuricemia have identified key susceptibility loci involved in uric acid transport and purine metabolism: Primary transporters include GLUT9 (*SLC2A9*) and URAT1 (*SLC22A12*), which mediate uric acid reabsorption. Gain-of-function variants in these genes enhance reabsorption, reduce excretion, and consequently elevate serum uric acid levels. BCRP, encoded by *ABCG2*, is a key secretory transporter; loss-of-function variants impair renal and intestinal uric acid excretion. Sites such as *PDZK1*, *GCKR*, and *HNF4A* indirectly influence uric acid levels by regulating transporter expression or activity, further highlighting the central role of these genetic variants in hyperuricemia susceptibility and progression. Relevant core genes and their major functional variants are summarized in Table 1.

**TABLE 1:** Core susceptibility genes and their encoding proteins.

Gene	Coding protein	Position	Functional mechanism	Most relevant SNPs	Key references
SLC2A9	GLUT9	1. Renal tubular basement membrane (GLUT9a) and apical membrane of collecting ducts (GLUT9b) 2. Apical and basolateral membranes of intestinal epithelium	1. Mediates uric acid reabsorption into the bloodstream, uric acid synthesis, and regulates URAT1-mediated “uric acid-organic anion” exchange efficiency; its overexpression causes hyperuricemia 2. Mediates intestinal uric acid secretion; its deficiency results in abnormal uric acid excretion	rs16890979 rs6832439 rs13131257 rs737267 rs4529048 rs10805346 rs6449213 rs3775948	Dehghan et al. (2008) Voruganti et al. (2013) Sull et al. (2013) Voruganti et al. (2014) Takei et al. (2021)
ABCG2	BCRP	Renal, intestinal, and hepatic parietal terminals	Uric acid secretory protein that transports intracellular urate to the extracellular compartment, facilitating its excretion in the renal tubular lumen, intestine, and bile	rs2231142 (p.Q141K)	Yang et al. (2018), Yamagishi et al. (2010)
SLC22A12	URAT1	The apical membrane of the renal proximal tubule	The uric acid reabsorption core transporter mediates ∼90% of uric acid reabsorption and is a regulatory target for multiple uric acid-lowering drugs [15]	p.W258X (rs121907892) p.T217M rs147647315	Enomoto et al. (2002b) Tin et al. (2019)
PDZK1	PDZK1 scaffolding protein	It depends on interacting proteins and specific types of cell	Regulates the function of uric acid transport proteins (e.g., ABCG2, URAT1, OAT4, NPT1), indirectly affecting uric acid metabolism	-	-
GCKR	Glucokinase regulatory protein (GCKR)	Liver	Indirectly affects purine metabolism via glucose regulation, thereby influencing uric acid production; also involved in renal uric acid excretion through lipid metabolism effects	rs780094 rs1260326	Wang et al. (2012), Ho et al. (2022)
HNF4A	Hepatocyte nuclear factor 4 alpha	Liver	Indirectly affects uric acid metabolism by regulating liver function and modulates the expression of uric acid transport-related genes (e.g., SLC2A9, PDZK1, MAF)	rs6031598 p.Thr139Ile	Nakatochi et al. (2019) Tin et al. (2019)

#### 3.1.1 Core genes for uric acid excretion

##### 3.1.1.1 SLC2A9

The *SLC2A9* gene was first cloned and identified in 2000, encoding the glucose transporter GLUT9, which is highly expressed in the liver and kidneys (Phay et al., 2000). In 2007, a GWAS first identified a single-nucleotide polymorphism (SNP) within *SLC2A9* as strongly associated with serum uric acid levels: individuals carrying the rare rs6855911 allele exhibited significantly lower serum uric acid levels than those with the common allele (Li et al., 2007). Subsequent studies confirmed that *SLC2A9* variants explain 1.7%–5.3% of serum uric acid variation in the Croatian population (Vitart et al., 2008). Further studies showed that common allele nearly doubles the risk of hyperuricemia (Wallace et al., 2008). Large-scale cohort studies in Framingham and Rotterdam further demonstrated that rs16890979 is significantly associated with serum uric acid across diverse ethnic populations (Dehghan et al., 2008). Subsequent studies in European, African American (Charles et al., 2011), Mexican American (Voruganti et al., 2013), Korean (Sull et al., 2013); American Indians (Voruganti et al., 2014), and Hispanic cohorts (Voruganti et al., 2015) consistently validated *SLC2A9* as the strongest genetic locus influencing serum uric acid, with multiple intronic and missense SNPs reaching genome-wide significance. Collectively, these findings indicate that *SLC2A9* is a stable genetic determinant of serum uric acid across racial populations.

Beyond genetic associations, functional studies have elucidated the biological mechanisms by which *SLC2A9* regulates uric acid homeostasis: alternative splicing generates two GLUT9 isoforms—GLUT9a (540 amino acids), localized to the basolateral membrane of proximal tubules, and GLUT9b (511 amino acids), localized to the apical membrane of collecting ducts. GLUT9a mediates trans-epithelial uric acid reabsorption, while the function of GLUT9b remains incompletely defined (Mandal and Mount, 2015). In African clawed frog oocytes, GLUT9 acts as a high-capacity, low-affinity uric acid transporter whose activity is inhibited by benzbromarone (Vitart et al., 2008). GLUT9 overexpression enhances uric acid reabsorption, directly promoting hyperuricemia development; Additionally, GLUT9-mediated fructose/glucose transport promotes hepatic uric acid production by activating the pentose phosphate pathway. In renal tubules, GLUT9 also regulates lactate concentration, thereby modulating URAT1-mediated uric acid exchange; however, this mechanism exacerbates hyperuricemia during purine loading or renal ischemia (Li et al., 2007).

In summary, *SLC2A9*/GLUT9 maintains uric acid homeostasis through three mechanisms: GLUT9a-mediated direct reabsorption, metabolic stimulation of hepatic uric acid synthesis, and microenvironmental regulation of URAT1 activity via lactate. Variants or abnormal expression of *SLC2A9* significantly alter serum uric acid levels, playing a pivotal role in the pathogenesis of hyperuricemia.

##### 3.1.1.2 ABCG2

The *ABCG2* gene was first identified in 1998 in the multidrug-resistant breast cancer cell line MCF-7/AdrVp. It encodes the ATP-binding cassette transporter BCRP, which mediates ATP-dependent efflux of multiple substrates, thereby contributing to multidrug resistance (Doyle et al., 1998). A 2008 GWAS highlighted that the missense variant rs2231142 (p.Q141K) in *ABCG2* is strongly associated with serum uric acid levels and gout risk (Dehghan et al., 2008). Subsequent functional studies confirmed that this variant reduces BCRP-mediated uric acid efflux, thereby elevating serum uric acid concentrations (Woodward et al., 2009). Large-scale population studies consistently validated the association between rs2231142 and hyperuricemia across diverse ethnicities. Significant associations were observed in European, African, Mexican, and Native American populations (Zhang et al., 2013), and East Asian populations (Yang et al., 2018; Yamagishi et al., 2010). Notably, in East Asians, rs2231142 ranks among the strongest genetic risk loci for hyperuricemia, with particularly high prevalence in China’s southeastern coastal regions. Additionally, rs2054576 and rs2725220 have also been confirmed as associated with hyperuricemia in a Korean cohort (Son et al., 2017; Sull et al., 2014). Collectively, these findings establish *ABCG2* as a core genetic determinant of hyperuricemia across populations.

Functionally, BCRP serves as a high-capacity uric acid efflux transporter expressed in the apical membranes of renal proximal tubules, intestinal epithelial cells, and hepatocytes. Under physiological conditions, it promotes ATP-dependent uric acid secretion into urine, bile, and the intestinal lumen to maintain uric acid homeostasis. Loss-of-function variants significantly reduce transport activity; for example, the p.Q141K variant decreases uric acid excretion by approximately 53% (Mandal and Mount, 2015). Impaired BCRP function in the kidney reduces tubular uric acid excretion, while decreased BCRP activity in the intestine and liver diminishes extra-renal uric acid clearance and increases renal uric acid load, collectively promoting the development of hyperuricemia.

In summary, *ABCG2*/BCRP is a key efflux transporter regulating systemic uric acid levels. Genetic variants such as p.Q141K significantly impair its function, constituting a major risk factor for hyperuricemia pathogenesis.

##### 3.1.1.3 SLC22A12

In 2002, research identified URAT1, encoded by the *SLC22A12* gene, as the primary renal uric acid transporter (Enomoto et al., 2002b). URAT1 localizes to the apical membranes of renal proximal tubule cells, and mediates uric acid reabsorption via anion exchange. Its activity depends on the chloride gradient but is independent of sodium cotransport, membrane potential, and pH (Mandal and Mount, 2015). URAT1 exchanges uric acid with organic anions such as lactate and nicotinate, while pyrazino-1,2,3-tricarboxylic acid significantly enhances uric acid uptake. This physiological function also makes URAT1 an important pharmacological target. Uricosuric agents like febuxostat reduce serum uric acid levels by inhibiting URAT1, thereby decreasing reabsorption and promoting excretion. Conversely, loss-of-function mutations in *SLC22A12* (e.g., p.W258X, p.T217M) cause idiopathic renal hypouricemia (Mandal and Mount, 2015).

GWAS further confirmed *SLC22A12*’s contribution to uric acid regulation: a large cross-ethnic GWAS (N = 457,690) identified 183 loci explaining 17% of serum uric acid heritability, with core variants in *SLC2A9*, *ABCG2*, and *SLC22A12* collectively contributing approximately 5% (Tin et al., 2019). In individuals of African ancestry, genome-wide significant variants in *SLC2A9* (rs7683856, rs6838021) and *SLC22A12* (rs147647315) collectively explained 4.3% of serum uric acid variation. These findings establish *SLC22A12* as a key uric acid transporter and an important contributor to hyperuricemia susceptibility across populations.

##### 3.1.1.4 PDZK1

The *PDZK1* gene encodes a scaffold protein containing a PDZ domain that regulates the activity of multiple uric acid transporters. As a binding chaperone for URAT1, *PDZK1* enhances URAT1-mediated uric acid uptake (Anzai et al., 2004). It also influences *ABCG2* function by regulating its expression, activity, subcellular localization, and specific signaling pathways (Chen et al., 2018). *PDZK1* interacts with uric acid transporters such as URAT1, OAT4, and NPT1 to form bidirectional transport system, achieving functional coupling between different transporters (Wright et al., 2010). By organizing and regulating these transporter networks, *PDZK1* plays a crucial role in uric acid homeostasis. Loss of *PDZK1*activity disrupts the function of multiple transporters, indirectly impairing uric acid clearance and promoting the development of hyperuricemia (Higashino et al., 2016).

#### 3.1.2 Metabolic regulatory genes

##### 3.1.2.1 GCKR

The *GCKR* gene encodes the glucose kinase regulator protein, which modulates glucose metabolism by interacting with glucose kinase and inhibiting its activity. In 2009, a study first reported that the rs780094 polymorphism in *GCKR* was associated with serum uric acid levels (P = 1.46 × 10^−9^), with a more pronounced effect in males (Kolz et al., 2009). Subsequently, research confirmed this association in an Asian cohort, linking rs780093 and rs780094 to hyperuricemia (Wang et al., 2012). Follow-up studies identified gender-specific effects: rs1260326 and rs780094 showed stronger associations in girls than in boys (Ho et al., 2022).

Given the close relationship between glucose and purine metabolism, *GCKR* can indirectly influence purine metabolism and uric acid production by regulating glucose metabolism. *GCKR* variants are also associated with lipid metabolism, which in turn affects renal uric acid excretion. For example, the rs1260326 variant alters *GCKR* expression, disrupting glucose metabolism and elevating uric acid levels. Furthermore, *GCKR* gene variants may affect uric acid transporters such as *ABCG2* and *SLC2A9*, thereby simultaneously influencing uric acid production and excretion (Tanikawa et al., 2019; Mandal and Mount, 2015).

##### 3.1.2.2 HNF4A

The *HNF4A* gene (rs6031598) is primarily expressed in the liver and was first associated with serum uric acid levels (P = 2.90 × 10^−8^) via GWAS in 2019 (Nakatochi et al., 2019). *HNF4A* directly influences glucose metabolism by regulating liver function, indirectly affecting purine metabolism, and ultimately impacting uric acid production. Subsequently, it was confirmed that the p.Thr139Ile variant in *HNF4A* enhances transcriptional activity at the *ABCG2* promoter, increasing its expression and promoting uric acid excretion (Tin et al., 2019). Recent evidence further indicates that *HNF4A* regulates the expression of multiple uric acid transporter genes, including *SLC2A9*, *PDZK1*, and MAF, participating in multi-pathway regulation of uric acid homeostasis. eQTL analysis and functional validation confirmed that the transcription factor encoded by *HNF4A* is expressed in the renal proximal tubule and regulates the expression of the uric acid transporter gene (Leask et al., 2020). These findings establish *HNF4A* as a key transcription factor in uric acid metabolism, regulating uric acid production and excretion.

### 3.2 Population heterogeneity in genetic effects

Numerous GWAS studies indicate significant heterogeneity in the genetic regulation of serum uric acid levels across different populations. At the racial/ethnic level, a genome-wide significant locus (p = 1.24 × 10^−4^) strongly associated with serum uric acid levels was identified on chromosome 11p15.4 in African Americans. Fine-mapping revealed rs2855126 upstream of *HBG1* as the key variant site (Shriner et al., 2015). In Hispanic children, multiple zinc finger protein genes (e.g., *ZNF446*, *ZNF584*, *ZNF132*) located on 19q13 were significantly associated with uric acid clearance, with rs2033711 in *ZNF446* reaching genome-wide significance (p < 8 × 10^−8^) (Chittoor et al., 2017).

Among Han Chinese males, the rs671 polymorphism in the *ALDH2* gene was significantly associated with serum uric acid levels (p = 1.2 × 10^−10^). Although it accounts for only a small proportion of phenotypic variation (0.21%–1.95%), this variant is scarce in European and African populations (Zhang et al., 2018), highlighting pronounced racial specificity.

Furthermore, researchers confirmed in a Korean cohort that the p. W258X variant (rs121907892) in the *SLC22A12* gene accounts for 8.1% of interindividual variation in serum uric acid levels. This functional variant is particularly prevalent in East Asian populations (Cho et al., 2020; Im et al., 2020).

Simultaneously, genetic effects on uric acid metabolism exhibit gender heterogeneity. A GWAS of 28,141 Europeans revealed that the rs734553 locus in the *SLC2A9* gene exerted a significantly more substantial effect on reducing serum uric acid levels in females (β = −0.397, P = 1.05 × 10^−192^) than in males (β = −0.220, P = 1.13 × 10^−41^) (Kolz et al., 2009). Subsequent studies further confirmed that *SLC2A9*/GLUT9-associated variants generally exerted a more substantial influence on serum uric acid levels in females, whereas *ABCG2* gene variants had a more pronounced effect on male serum uric acid levels (Choi et al., 2010). Mechanistic studies suggest this sex difference may relate to estrogen’s protective effects: Estrogen upregulates the expression of uric acid transporters, such as *ABCG2* and *SLC2A9*, as well as key transcription factors, such as HNF4α, thereby enhancing renal uric acid excretion and inhibiting uric acid reabsorption (Halperin Kuhns and Woodward, 2020).

### 3.3 Gene-environment interaction

Genetic susceptibility and environmental exposure exhibit significant interactive effects in the development of hyperuricemia. Dietary studies indicate that *LRP2* rs2544390 is associated with increased serum uric acid levels and hyperuricemia in Japanese populations, with a more pronounced effect among frequent drinkers. Conversely, opposite racial differences are observed in European and Maori/Pacific Islander populations (Hamajima et al., 2012; Rasheed et al., 2013). High fructose intake elevates serum uric acid by promoting hepatic uric acid production and inhibiting *SLC2A9*-mediated renal excretion, with varying effects across ethnic groups (Batt et al., 2014). Non-additive interactions between specific metabolic genes (e.g., *GCKR* rs780094, *A1CF* rs10821905) with alcohol intake can further promote uric acid synthesis and weaken the protective effects of lipid metabolism (Rasheed et al., 2017). Multicohort Mendelian randomization studies also support a causal association between alcohol intake and hyperuricemia (Jee et al., 2019). Recent diet-polygenic risk score (PRS)-stratified studies indicate that individuals with high genetic risk experience an exponential increase in hyperuricemia risk with elevated intake of red and processed meat (Lee and Shin, 2024), suggesting the need for personalized nutritional management in high-risk populations.

Drug-gene interactions also influence uric acid homeostasis. Thiazide diuretic-induced serum uric acid elevation exhibits distinct pathogenic genes across populations: African Americans primarily involve *LUC7L2*, *COX18*, and *ANKRD17*, while Caucasians are dominated by *GRIN3A* (Vandell et al., 2014). A Finnish cohort further identified *VEGFC*, *BRINP3*, and *PADI4* loci as associated with thiazide-related hyperuricemia risk (Ala-Mutka et al., 2018). In contrast, metformin reduces uric acid via the *AMPK* pathway (Dai et al., 2024), while the *SLC2A9* rs938564 variant enhances the uric acid reabsorption-promoting effect of thiazides (Asiimwe et al., 2024). Furthermore, vitamin B6 metabolic dysfunction and altered gut microbiota may promote hyperuricemia development by activating the S1P–S1PR3 inflammatory axis, suggesting the potential importance of microbe–host metabolic interactions (Liu X. et al., 2024).

Lifestyle factors also modulate the expression of genetic risk. Unhealthy lifestyles significantly elevate hyperuricemia risk, with PRS exhibiting a dose-dependent relationship with hyperuricemia risk. Although no significant statistical interaction between genetics and lifestyle was observed, stratified analysis revealed an additive effect: individuals with high PRS experienced exponentially increased hyperuricemia risk under unhealthy lifestyles, suggesting that healthy lifestyles may partially mitigate the metabolic burden of genetic susceptibility (Takase et al., 2024).

Collectively, environmental exposures such as diet, medication, and lifestyle, along with genetic factors, jointly determine individual hyperuricemia risk, exhibiting both population-specific variations and additive effects. Therefore, implementing precision nutrition and personalized lifestyle interventions among high-risk populations is crucial.

## 4 Mechanistic insights linking GWAS findings to pathophysiology

**FIGURE 1:**
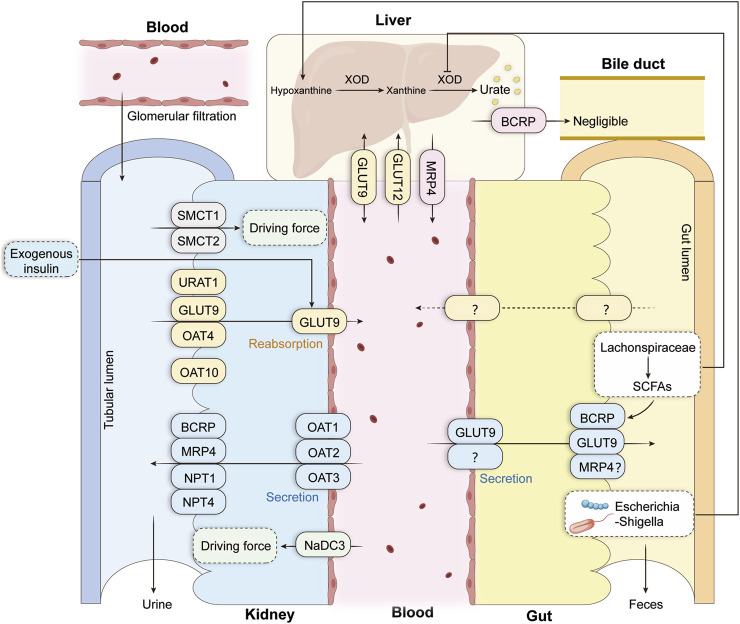
Mechanism of uric acid metabolism. Uric acid is generated in the liver by XOD catalysis and transported to tissues via uric acid transporter proteins, which are mainly excreted via the kidney (70%) and intestine (30%). URAT1 is the most important transporter protein for renal UA reabsorption, and BCRP is an important uric acid secretion transporter protein in the intestine. Uric acid homeostasis is also regulated by various metabolic factors, such as exogenous insulin to promote renal GLUT9 reabsorption, the intestinal microorganisms Escherichia-Shigella to promote uric acid production by affecting purine metabolism. Lachnospiraceae to promote the intestinal BCRP to transport UA while inhibiting hepatic XOD activity (This figure was adapted from the concept presented by Wen et al. (2024). Abbreviations: UA, uric acid; XOD/XO, xanthine oxidase/dehydrogenase; URAT1, urate transporter 1; GLUT9, glucose transporter 9; OAT1/2/3/4, organic anion transporters; BCRP, breast cancer resistance protein (ABCG2); MRP4, multidrug resistance-associated protein 4; SMCT1/2, sodium-coupled monocarboxylate transporters 1/2; NPT1/4, sodium phosphate transporters 1/4; SCFAs, short-chain fatty acids.

The production and excretion of uric acid are jointly regulated by genetic, metabolic, and environmental factors, resulting in a highly complex pathogenesis. In recent years, large-scale GWAS studies have significantly deepened our understanding of these processes, particularly in identifying key genes and regulatory networks involved in purine metabolism and UA homeostasis. As shown in Figure 1, purines are oxidized to uric acid in the liver by xanthine oxidase (XOD). In contrast, systemic uric acid levels are maintained through coordinated action across multiple organs, including hepatic synthesis, renal reabsorption and excretion, and intestinal clearance.

### 4.1 Renal excretion

Following glomerular filtration, uric acid undergoes two tightly regulated processes in the proximal tubule: secretion and reabsorption. Circulating uric acid enters epithelial cells primarily via transporters OAT1 (Sekine et al., 1997), OAT2 (Enomoto et al., 2002a; Sato et al., 2010), and OAT3 (Bakhiya et al., 2003). Subsequently, uric acid is secreted into the tubular lumen via NPT1 (Iharada et al., 2010; Uchino et al., 2000), NPT4 (Jutabha et al., 2010), BCRP (Woodward et al., 2009), and MRP4 (Van Aubel et al., 2005). Conversely, reabsorption is primarily mediated by URAT1 (Enomoto et al., 2002a; Lu et al., 2014; Nakanishi et al., 2013; Sato et al., 2011), OAT4 (Hagos et al., 2007), OAT10 (Bahn et al., 2008) and basolateral GLUT9a (Preitner et al., 2009). SMCT1/SMCT2 (Gopal et al., 2004; Lu et al., 2013; Gopal et al., 2007) provides exchange substrates for reabsorption, while NaDC3 (Pajor et al., 2001) indirectly regulates uric acid flux through interactions with OATs. These processes collectively maintain the kidney’s fine-tuned regulation of uric acid.

### 4.2 Intestinal, hepatic, and systemic regulation

Approximately one-third of uric acid is excreted via the gut (Liu et al., 2023), with ABCG2 playing a dominant role (Hosomi et al., 2012; Ichida et al., 2012). The MRP series provides auxiliary excretion (Hilgendorf et al., 2007), and GLUT9 may also contribute to intestinal uric acid flux (Debosch et al., 2014). The liver is the primary organ for uric acid synthesis. Basolateral membrane GLUT9 and GLUT12 in hepatocytes regulate uric acid output into the circulation, while biliary excretion is negligible (Hosomi et al., 2012).

Systemic metabolic signals further influence uric acid handling: insulin promotes renal tubular reabsorption and reduces uric acid clearance (Ter Maaten et al., 1997; Mount et al., 2018); impaired glycolysis (e.g., GA3PDH downregulation) enhances uric acid production (Tabák et al., 2009). Gut microbiota participate in regulation via purine metabolism or short-chain fatty acid pathways: increased *Escherichia* coli–Shigella abundance correlates positively with serum uric acid (Zhang W. et al., 2021), while phyla such as the Bacteroidetes can reduce serum uric acid by upregulating ABCG2, inhibiting hepatic XOD, and alleviating diet-induced stress (Xi et al., 2000; Sanna et al., 2019; Meehan and Beiko, 2014; Lou et al., 2024).

## 5 Mechanisms of TCM in regulating uric acid homeostasis

**FIGURE 2:**
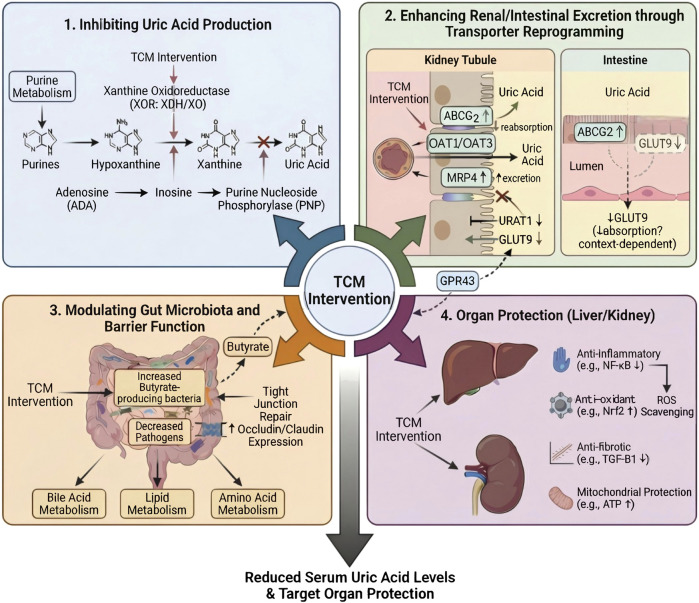
Comprehensive Mechanism of TCM in Improving Hyperuricemia. TCM interventions primarily exert effects through four complementary pathways: (1) Inhibition of uric acid production, mainly by regulating key enzymes in the purine metabolic pathway (xanthine oxidoreductase, including XDH/XO; adenosine deaminase; purine nucleoside phosphorylase). (2) Enhancing renal and intestinal uric acid excretion through transporter reprogramming, characterized by downregulation of URAT1 and GLUT9, and upregulation of ABCG2, OAT1/OAT3, and MRP4. ABCG2 primarily mediates intestinal uric acid excretion, while intestinal GLUT9 function depends on its subtype and environment. (3) Modulate gut microbiota and enhance intestinal barrier integrity, including enrichment of butyrate-producing bacteria, reduction of pathogenic bacteria, restoration of tight junctions, and regulation of bile acid, lipid, and amino acid metabolism. (4) Protect the liver and kidneys through anti-inflammatory, antioxidant, anti-fibrotic, and mitochondrial protective effects. These mechanisms collectively reduce serum uric acid levels and protect target organs. Abbreviations: XOR, xanthine oxidoreductase; XDH, xanthine dehydrogenase; XO, xanthine oxidase; ADA, adenosine deaminase; PNP, purine nucleoside phosphorylase; ABCG2, ATP-binding cassette subfamily G member 2; OAT1/OAT3, organic anion transporter 1/3; MRP4, multidrug resistance-associated protein 4; URAT1, urate transporter 1; GLUT9, glucose transporter 9; GPR43, G-protein coupled receptor 43; ROS, reactive oxygen species.

GWAS has identified key genetic loci and associated genes influencing serum uric acid levels, providing crucial insights into the molecular basis of uric acid homeostasis. Since uric acid homeostasis is jointly regulated by multiple pathways—including hepatic synthesis, renal excretion, intestinal efflux, and systemic metabolism—therapeutic strategies often require targeting multiple regulatory nodes. TCM, characterized by multi-component, multi-target, and systemic regulation, can exert effects across multiple levels—including uric acid production, renal and intestinal transport, metabolic regulation, immune and inflammatory responses, and gut microbiota—thereby achieving comprehensive control of uric acid homeostasis. This section reviews the mechanisms by which TCM regulates uric acid homeostasis at three levels: TCM formulas, botanical drugs, and active metabolites. An integrated mechanistic framework is illustrated in Figure 2, while the major therapeutic mechanisms of formulas, botanical drugs, and active metabolites are summarized in Tables 2–4, respectively.

**TABLE 2:** Mechanisms of TCM formulas in treating hyperuricemia.

Formula	Composition	Model	Induction method	Dose range	Minimum effective dose	Optimal dose	Negative control	Positive control	Duration	Mechanisms	Ref.
Wuling San	*Polyporus umbellatus* *Poria cocos* *Alisma orientale* *Cinnamomm cassia* *Atractylodes macrocephala*	Human adults	-	4.5 g twice daily	-	-	Placebo	-	4 weeks	↓URAT1,GLUT9 ↓NLRP3 inflammasome	Leong et al. (2024)
Ermiao San–related formulas	*Phellodendron amurense* *Atractylodes lancea* *Achyranthes bidentata* *Coix lacryma-jobi* var. *ma-yuen*	Rats	10% fructose water + potassium oxonate (500 mg/kg, i.p.) for 30 days	1.10, 1.29, 1.414 g/kg	1.10 g/kg	1.414 g/kg	Normal control	-	30 days	Regulation of metabolic pathways; improvement of metabolic dysfunction; hepatoprotective and renoprotective	Shan et al. (2021)
Simiao Wan	*Phellodendron amurense* *Atractylodes lancea* *Coix lacryma-jobi* var. *ma-yuen* *Achyranthes bidentata*	Kunming male mice	Potassium oxonate (300 mg/kg, i.p.) + xanthine (500 mg/kg, p.o.) for 14 days	0.31, 0.62, 1.23 g/kg	0.31 g/kg	1.23 g/kg	Normal control	Allopurinol 10 mg/kg	14 days	↓XOD,XDH ↓GLUT9,URAT1,OAT4 ↑ABCG2,OAT1; anti-inflammatory; anti-apoptotic; anti-fibrotic	Zeng et al. (2024)
Simiao San	*Phellodendron amurense* *Atractylodes lancea* *Coix lacryma-jobi* var. *ma-yuen* *Achyranthes bidentata*	SD male rats	Potassium oxonate 150 mg/kg (in 0.5% CMC-Na + 0.1 M sodium acetate)	100, 200, 400 mg/kg	100 mg/kg	400 mg/kg	Normal control	Allopurinol 10 mg/kg	5 days	↓XOD ↓URAT1,GLUT9 ↑OAT1,OCT2 ↑beneficial gut microbiota	Qian and Shen (2024)
Simiao San	*Phellodendron amurense* *Atractylodes lancea* *Coix lacryma-jobi* var. *ma-yuen* *Achyranthes bidentata*	Kunming mice	Potassium oxonate 300 mg/kg (i.p.) + xanthine 500 mg/kg (p.o.) for 7 days	4.55, 9.1, 18.2 g/kg/d	4.55 g/kg/d	18.2 g/kg/d	Normal control	Allopurinol 5 mg/kg/d	7 days	↓URAT1 GLUT9 ↑OAT1 ↓ NLRP3 inflammasome	Zhang et al. (2023)
Fangji Huangqi Tang	*Stephania tetrandra* *Astragalus mongholicus* *Atractylodes macrocephal* *Glycyrrhiza uralensis*	Kunming male mice; HK-2 cells	Potassium oxonate 250 mg/kg (i.p.) for 7 days	10,920, 5,460, 2,730 mg/kg	2,730 mg/kg	10,920 mg/kg	Normal control	Allopurinol 5 mg/kg	7 days; 24 h	↑OAT1, OAT3 ABCG2, PDZK1 ↓ IL-1β, NF-B,*LRRK1*, EGFR	Wang et al. (2020)

Dose ranges are presented as administered in each original experiment. “Minimum effective dose” refers to the lowest dose reported to exert significant antihyperuricemic activity. “Optimal dose” refers to the strongest therapeutic effect within the tested range. Abbreviations: XOD, xanthine oxidase; XDH, xanthine dehydrogenase; URAT1, urate transporter 1; GLUT9, glucose transporter 9; OAT, organic anion transporter; OCT2, organic cation transporter 2; ABCG2, ATP-binding cassette subfamily G member 2; PDZK1, PDZ-domain-containing 1; NF-κB, nuclear factor-κB; NLRP3, NOD-like receptor family pyroptosis protein 3; EGFR, epidermal growth factor receptor.

**TABLE 3:** Mechanisms of botanical drugs in treating hyperuricemia.

Botanical drugs	Model	Induction method	Dose range	Minimum effective dose	Optimal dose	Negative control	Positive control	Duration	Mechanisms	Ref.
*Phellodendron amurense*	ICR mice	i.p. potassium oxonate (300 mg/kg) + oral xanthine (300 mg/kg) for 10 days	200, 400, 800 mg/kg	200 mg/kg	800 mg/kg	Normal control	Allopurinol 20 mg/kg	10 days	↓XOD ↓Cr, BUN ↓PI3K/Akt, TNF, NF-κB, IL-6	Xu et al. (2022)
*Polygonum capitatum*	Kunming mice	i.p. 100 mg/kg PO + oral hypoxanthine 500 mg/kg	WP: 175, 350, 700 mg/kg; EP: 115, 230, 460 mg/kg	WP 175 mg/kg EP 115 mg/kg	WP 700 mg/kg; EP 460 mg/kg	Normal control	Allopurinol 5 mg/kg; Benzbromarone 7.8 mg/kg	7 days	↓XOD ↓URAT1, GLUT9	Zhang et al. (2021)
*Clerodendranthus spicatus*	C57BL/6J mice	Oral adenine (75 mg/kg) + i.p. potassium oxonate (200 mg/kg) for 25 days	100, 200, 400 mg/kg	100 mg/kg	400 mg/kg	Normal control	Benzbromarone 50 mg/kg	25 days	↓XOD, PRPS ↓ URAT1, GLUT9 ↑ OAT1, ABCG2 ↓NLRP3, TLR4/MYD88; gut microbiota regulation	Wang et al. (2024)
	C57BL/6 male mice	i.p. potassium oxonate 300 mg/kg for 14 days	160 mg/mL	-	-	Normal control	Allopurinol 5 mg/kg	14 days	Gut microbiota regulation; improvement of metabolic disorder; protection of renal injury	Chen et al. (2023)
	SD rats	10% fructose water + 20% yeast diet for 28 days	5, 10 g/kg/d	5 g/kg/day	10 g/kg/day	Normal control	Benzbromarone 20 mg/kg/day	28 days	↑ABCG2; gut microbiota modulation	Zhu et al. (2023)
*Plantago Seed*	SD rats	Oral potassium oxonate 1.5 g/kg for 28 days	3.75 g/kg	-	-	Normal control	Benzbromarone 10 mg/kg	28 days	↓XOD ↓URAT1,GLUT9 ↑PPAR signaling ↓TNF-α, IL-6	Liu et al. (2024)
*Mulberry Bark*	Kunming mice	Oral PO (500 mg/kg) + adenine (300 mg/kg) for 14 days	100, 200, 400 mg/kg	100 mg/kg	400 mg/kg	Normal control	Allopurinol 10 mg/kg; Benzbromarone 10 mg/kg	14 days	↓XOD ↓URAT1,GLUT9 ↑ABCG2 ↓TNF-α,IL-1β,IL-6	Wang et al. (2025)
*Cichorium intybus*	SD rats; NRK-52E cells	Rats:PO potassium oxonate 1 g/kg + adenine 80 mg/kg + yeast extract 10 g/kg for 28 days + single LPS injection (0.2 mg/kg); Cells: UA 1200 μmol/L + LPS 1 μg/mL, 24 h	7.5, 15 g/kg	7.5 g/kg	15 g/kg	Normal control	Benzbromarone 30 mg/kg	28 days	↑ ABCG2 ↓IL-6,TNF-α, hs-CRP ↑lncRNA H19 ↓miR-21-3p	An et al. (2025)
Cichorium intybus	SD rats	PO potassium oxonate 750 mg/kg + yeast extract 10 g/kg for 28 days	5, 10 g/kg	5 g/kg	10 g/kg	Normal control	-	28天	↑Butyrate ↑ABCG2 ↑Claudin-1	Yang et al. (2025)
*Hovenia acerba*	KM mice	Oral potassium oxonate 100 mg/kg + xanthine 500 mg/kg for 7 days	78, 156, 312 mg/kg	78 mg/kg	312 mg/kg	Normal control	Allopurinol 10 mg/kg; Benzbromarone 10 mg/kg	7 days	↓XOD ↑ ABCG2, OAT1 ↓URAT1, GLUT9 ↓ATF3, CXCR4	Wang et al. (2025)
*Paeonia lactiflora*	KM mice; BRL-3A hepatocytes	Mice: i.p. oxonate 300 mg/kg + oral xanthine 500 mg/kg for 7 days; Cells: adenosine 0.5 mM, 24 h	Mice:150, 300, 600 mg/kg/day; Cells:50, 100, 200 μg/mL	Mice:150 mg/kg/day Cells:50 μg/mL	Mice: 600 mg/kg/day; Cells: 200 μg/mL	Normal control	Allopurinol 10 mg/kg/day; cells: allopurinol 10 μM	7 days	↓XOD ↓URAT1, GLUT9 ↑ OAT1, ABCG2	Du et al. (2024)
*Thlaspi arvense*	SD male rats	i.p. oxonate 350 mg/kg + oral yeast extract 10 g/kg for 21 days	3.5 g/kg	-	-	Normal control	Benzbromarone 4.5 mg/kg	14 days	↓XOD ↓URAT1,SLC4A4 ↑OAT1,OAT3,ABCG2 SLC2A9,SLC12A1,SLC16A ↑SOD,↓MDA	Ke et al. (2024)
*Gymnadenia conopsea*	Zebrafish	Oxonate + sodium xanthine, 25 h	100/200/400 μg/mL	100 μg/mL	400 μg/mL	Normal control	Allopurinol 10 μM	24 h	↓XOD ↓ROS,MDA,SOD	Chen T. et al. (2022)
*Astragalus mongholicus*	KM mice; CACO-2, HK-2 cells	Mice: adenine 200 mg/kg + oxonate 500 mg/kg for 2 weeks; Cells: UA 1000 μg/mL, 24 h	Mice: 130, 260, 520 mg/kg/day; Cells: 0.5, 1, 2 mg/mL; astragaloside-IV 20–40 μg/mL	Mice: 130 mg/kg/day; Cells: 1 mg/mL	Mice: 520 mg/kg/day; Cells: 2 mg/mL	Normal control	Allopurinol 10 mg/kg	Mice: 14 days; Cells: 24 h	↓URAT1,GLUT9 ↑ABCG2 ↓XOD	Zhang et al. (2023)
*Astragalus mongholicus*	C57BL/6J mice	i.p. potassium oxonate 300 mg/kg	1.54, 6.17 g/kg	1.54 g/kg	6.17 g/kg	Normal control	Allopurinol 5 mg/kg	14 days	Regulation of gut microbiota and lipid metabolism	Deng et al. (2023)
*Smilax china*	Chickens	High-calcium, high-protein diet for 14 days	10, 30, 90 mg/kg	10 mg/kg	90 mg/kg	Normal control	*Lysimachia christinae* 8 g/kg diet	5 days	↓XOD ↑BCRP, MRP4 ↓IL-6, TNF-α	Yan et al. (2024)
*Ilex cornuta*	Mice	Oral adenine 100 mg/kg/day + oxonate 250 mg/kg/day for 21 days	240, 480, 960 mg/kg	240 mg/kg	960 mg/kg	Normal control	Allopurinol 10 mg/kg	21 days	↓XOD ↑ABCG2, OAT1, OAT3 ↓ GLUT9 ↓NF-κB signaling, TNF-α, IL-1β ↓renal apoptosis	Zhou et al. (2025)

XOD, xanthine oxidase; URAT1, urate transporter 1; GLUT9, glucose transporter 9; OAT/OAT1/3, organic anion transporters; ABCG2, ATP-binding cassette G2; PRPS, phosphoribosyl pyrophosphate synthetase; SOD, superoxide dismutase; MDA, malondialdehyde; NF-κB, nuclear factor-κB; IL-1β, interleukin-1β; TNF-α, tumor necrosis factor-α; Cr, creatinine; BUN, blood urea nitrogen; UA, uric acid; hs-CRP, high-sensitivity C-reactive protein.

**TABLE 4:** Mechanisms of active metabolites in treating hyperuricemia.

Category	Active metabolites	Plant source	Extract type	Model	Induction method	Dose range	Minimum effective dose	Optimal dose	Negative control	Positive control	Duration	Mechanisms	Ref.
Phenolics	1-(4-hydroxy-2-methoxyphenyl)-2-(4-hydroxy-3,5-dimethyl- phenyl)butane-1,2,3-triol	*Reynoutria japonica*	80% ethanol-eluted fraction	ICR mice	i.p. PO 300 mg/kg	1.0, 0.5 g/kg	0.5 g/kg	1.0 g/kg	Normal control	Allopurinol 30 mg/(kg·day)	9 days	↓XOD; metabolic pathway modulation	Hu et al. (2023)
	Punicalagin	*Punica granatum*	Standard compound	Kunming mice	Oral adenine 200 mg/kg + PO 300 mg/kg for 2 weeks	100, 200, 300 mg/kg	100 mg/kg	300 mg/kg	Normal control	Benzbromarone 10 mg/(kg·day)	2 weeks	↓URAT1,GLUT9 ↑ABCG2,OAT1,GLUT9; ↓MAPK/NF-κB; gut microbiota modulation	Han et al. (2025)
	Saffron Petal Flavonoid Extract	*Crocus sativus*	Flavonoid extract	SD rats	Oral PO 1.5 g/kg for 2 weeks	0.085, 0.17,0.34 g/kg	0.085 g/kg	0.34 g/kg	Normal control	Allopurinol 0.02 g/(kg·day)	2 weeks	↑ABCG2 ↓URAT1,GLUT9 ↑SOD; ↓ MDA ↓NF-κB	Chen et al. (2025)
	Quercetin	*Various medicinal plants*	Standard compound	SD rats	Oral adenine 0.1 g/kg + PO 1.5 g/kg for 4 weeks	50, 100 mg/kg	50 mg/kg	100 mg/kg	Normal control	Febuxostat 5 mg/kg/day	4 weeks	↓GLUT9 ↓TLR4/NF-κB, IL-1β, TNF-α ↑SOD, GSH-Px ↓Bax, ↑Bcl- ↓GRP78, CHOP, p-PERK	Liu et al. (2025)
	Glycitein	*Lycium barbarum*	Standard compound	Zebrafish	200 μM PO + 10 μM sodium xanthine	25, 50, 100 μM	25 μM	100 μM	Normal control	Allopurinol 100 μM	-	↓XOD	​
	Linarin	*Chrysanthemum indicum*	Standard compound	ICR mice NRK-52E cells	Mice: i.p. PO 300 mg/kg + oral HX 300 mg/kg for 7 days Cells: adenosine 2.5 mM + XO 0.005 U/mL, 24 h	Mice:30, 60, 120 mg/kg/day Cells: 2.5, 10, 40 μM	30 mg/kg; 2.5 μM	120 mg/kg; 40 μM	Normal control	Benzbromarone 20 mg/kg/day; Febuxostat 10 mg/kg/day	14 days; 24 h	↓XOD ↓URAT1, GLUT9 ↑ ABCG2, OAT1, OAT3 ↑Nrf2/Keap1 ↓TLR4/NF-κB ↓ IL-6, TNF-α	Qian et al. (2025)
	Chrysanthemi Indici Flos-enriched flavonoid part	*Chrysanthemum indicum*	Flavonoid-enriched fraction	ICR mice	Weeks 1–4: oral adenine 100 mg/kg + PO 300 mg/kg; Weeks 5–6: adenine 100 mg/kg + PO 500 mg/kg	30, 60, 90 mg/kg/day	30 mg/kg	90 mg/kg	Normal control	Allopurinol 10 mg/kg/day	6 weeks	↓XOD, ADA ↓URAT1, GLUT9, OAT4; ↑OAT1, OAT3, ABCG2	Jiao et al. (2024)
	Puerarin	*Pueraria montana* var. *lobata*	Standard compound	C57BL/6J mice	Diet: adenine 0.25% + PO 5% for 14 days	100 mg/kg/day	-	-	Normal control	Benzbromarone 40 mg/kg/day	3 weeks	↓XOD,ADA ↑ABCG2,OAT1 ↑ZO-1,Occludin ↑microbiota improvement	Li et al. (2025)
Glycopeptides	Ganoderma lucidum polysaccharide peptide	*Ganoderma lucidum*	Polysaccharide-peptide extract	ICR mice; HK-2 cells	Mice: i.p. PO 600 mg/kg or PO 300 mg/kg + HX 300 mg/kg for 2 weeks; Cells: adenosine 2.5 mM 30 h + XOD 8 h	Mice:50, 100 mg/kg/day Cells: 6.25, 25, 100 μg/mL	50 mg/kg 6.25 μg/mL	100 mg/kg 100 μg/mL	Normal control	Allopurinol 5 mg/kg; 100 μM	3 weeks; 62 h	↓ADA ↓GLUT9, ↑OAT1	Lin et al. (2022)
Saponins	Dioscin	*Dioscorea nipponica*	Standard compound	Kunming mice	Oral PO 200 mg/kg for 42 days	50 mg/kg/day	-	-	Normal control	-	42 days	Metabolic regulation; renal protection	Tan et al. (2021)
	Dioscin, Diosgenin	*Dioscorea nipponica*	Standard compound	SD rats	Oral PO 500 mg/kg + oral HX 500 mg/kg + hypoxia induction	10, 30, 90 mg/kg/day	10 mg/kg	90 mg/kg	Normal control	Allopurinol 10 mg/kg/day	6 days	↑EPHX2 ↓IL-1β, IL-6, TNF-α	Dang et al. (2024)
	WW-60%-1	*Clematis chinensis*	60% ethanol-eluted saponin fraction	Mice	20% yeast diet + s.c. PO 30 mg/kg + oral HX 30 mg/kg	12.5, 25, 50 mg/kg	12.5 mg/kg	50 mg/kg	Normal control	Allopurinol 5 mg/kg	14 days	↓ NF-κB, PI3K-Akt ↓oxidative stress	Liu et al. (2025)
Glycosides	Total Glucosides of Herbaceous Peony Flower	*Paeonia lactiflora*	Total glucosides	Wistar rats	Oral adenine 100 mg/kg + EH 250 mg/kg for 23 days	200 mg/kg/day	-	-	Normal control	Allopurinol 42 mg/kg/day	23 days	↓URAT1, GLUT9, ↑OAT1 ↓XOD ↓MCP-1, TNF-α	Kang et al. (2020)
Alkaloids	Berberine	*Phellodendron amurense* *Coptis chinensis*	Standard compound	ICR mice	i.p. PO 300 mg/kg + oral HX 300 mg/kg for 10 days	50, 100 mg/kg/day	50 mg/kg	100 mg/kg	Normal control	Benzbromarone 5 mg//kg/day	10 days	↓NLRP3,ASC,Caspase1,IL-1β,IL-18 ↓URAT1 ↓BUN, CRE	Li et al. (2021)
	9-Hydroxy-8-oxypalmatine	*Phellodendron amurense*	Synthetic metabolite (standard compound)	Kunming mice	.p. PO 300 mg/kg + oral HX 300 mg/kg for 7 days	5, 10, 20 mg/kg/day	5 mg/kg	20 mg/kg	Normal control	Febuxostat 5 mg//kg/day	7 days	↓XOD, ADA ↓ URAT1, GLUT9, ↑OAT1 ↓ NLRP3	Wu et al. (2024)
Terpenoids	Geniposide derivatives	*Gardenia jasminoides*	Chemically synthesized derivative	Kunming mice; HK-2 cells	Mice: oral adenine 200 mg/kg + PO 500 mg/kg for 2 weeks; Cells: UA 800 μM for 48 h	5–20 mg/kg; 12.5–100 μM	5 mg/kg; 12.5 μM	20 mg/kg; 100 μM	Normal control	Allopurinol 20 mg/kg/day	2 weeks; 48 h	↓XOD ↓TNF-α, IL-1β ↓α-SMA, Collagen I	Chen J. et al. (2022)
Polysaccharides	Polygonati Rhizoma polysaccharide	*Polygonatum sibiricum*	Polysaccharide extract	C57BL/6J mice; HK-2 cells	Mice: oral PO 250 mg/kg + oral HX 250 mg/kg for 2 weeks; Cells: XO/XA co-incubation 24 h	125, 250, 500 mg/kg/day 500 μg/mL	125 mg/kg	500 mg/kg	Normal control	Allopurinol 5 mg/kg/day	2 weeks; 24 h	↓XOD, ADA ↓URAT1, ↑OAT1, OAT3 ↓ROS	Zhang et al. (2024)
Oligosaccharides	Coix Seed Oligosaccharides	*Coix lacryma-jobi* var. *ma-yuen*	Oligosaccharide extract	Mice; HK-2 cells	Mice: i.p. PO 300 mg/kg + oral HX 300 mg/kg for 3 weeks; Cells: adenosine + XO for 32 h	50, 100, 200 mg/kg 100, 200, 400 μg/mL	50 mg/kg; 100 μg/mL	200 mg/kg; 400 μg/mL	Normal control	Allopurinol 5 mg/kg	3 weeks; 24 h	↓XOD ↓URAT1, GLUT9 ↑OAT1, ABCG2, OCT1 ↓IL-6/JAK2/STAT3	Wu et al. (2025)

XOD, xanthine oxidase; ADA, adenosine deaminase; URAT1, urate transporter 1; GLUT9, glucose transporter 9; ABCG2, ATP-binding cassette sub-family G member 2; OAT, organic anion transporter; OAT1/3, organic anion transporter 1/3; OCT1, organic cation transporter 1; PO, potassium oxonate; HX, hypoxanthine; UA, uric acid; SOD, superoxide dismutase; MDA, malondialdehyde; IL, interleukin; TNF-α, tumor necrosis factor-α; NF-κB, nuclear factor kappa B; JAK2, Janus kinase 2; STAT3, signal transducer and activator of transcription 3; MCP-1, monocyte chemoattractant protein-1; α-SMA, alpha-smooth muscle actin.

Beyond modern pharmacological research, traditional Chinese medical theory offers unique insights into the disease’s pathogenesis. Although classical texts do not explicitly name “hyperuricemia,” its asymptomatic phase and lack of specific manifestations led traditional practitioners to classify it under categories like “pre-disease” or “turbid-stasis.” The core pathogenesis of hyperuricemia lies in spleen-kidney deficiency, with dampness, heat, stasis, and toxins as secondary factors. Damp-turbidity pervades the entire disease course as the primary pathological product. Prolonged accumulation of damp-turbidity can transform into heat, obstruct meridians, and lead to clinical manifestations such as joint redness, swelling, heat, pain, and renal impairment. TCM treatment emphasizes “addressing symptoms in acute cases and root causes in chronic cases.” For asymptomatic hyperuricemia, the primary approach focuses on dispelling dampness and resolving turbidity, while concurrently strengthening the spleen and nourishing the kidneys to prevent the accumulation of damp-turbidity and its subsequent pathological progression.

### 5.1 TCM formulas

#### 5.1.1 Wuling San

Wuling San originates from the *Treatise on Cold Damage (Shang Han Lun)*, created by the Eastern Han physician Zhang Zhongjing. It is a classic diuretic and dampness-draining formula in traditional Chinese medicine, widely used for various metabolic disorders. The formula includes:*Polyporus umbellatus* (Pers.) Fries (Polyporaceae), *Poria cocos* (Schwein.) F.A. Wolf (Polyporaceae), *Alisma orientale* (Sam.) Juz. (Alismataceae), *Cinnamomum cassia* (L.). J. Presl (Lauraceae), *Atractylodes macrocephala* Koidz. (Asteraceae). Research indicates that Wuling San inhibits the NLRP3 inflammasome, ameliorates renal inflammatory responses, and reduces serum uric acid by downregulating URAT1 and GLUT9 expression while suppressing renal tubular reabsorption. Additionally, this formula demonstrates specific protective effects against renal injury (Leong et al., 2024).

#### 5.1.2 Ermiao San–related formulas

Ermiao San was first documented in Zhu Danxi’s Ming Dynasty text *Danxi’s Heart Methods (Danxi Xinfa)*. Composed of *Atractylodes lancea* (Thunb.) DC. (Asteraceae) And *Phellodendron amurense* Rupr. (Rutaceae), It clears heat, dries dampness, and promotes tendon relaxation and meridian circulation. Subsequent physicians developed Sanmiao San (adding *Achyranthes bidentata* Bl. (Amaranthaceae) and Simiao San (further adding *Coix lacryma-jobi* var. *ma-yuen* (Romanet du Caillaud) Stapf (Poaceae)), both used for bi syndrome caused by damp-heat descending. For narrative convenience, this study collectively refers to these formulas as “Ermiao San–related formulas.” These formulas regulate energy and purine metabolism pathways, improve metabolic disorders, and protect liver and kidney function. Among them, Simiao San demonstrates the most pronounced effects on the tricarboxylic acid cycle and purine metabolism (Shan et al., 2021). Their mechanisms include: inhibiting xanthine oxidase/dehydrogenase (XOD/XDH) activity to reduce uric acid production; downregulating renal reabsorption transporters URAT1 and GLUT9 while upregulating excretion transporters ABCG2 and OAT1 to enhance uric acid excretion; improving gut microbiota composition by increasing butyrate-producing bacteria (e.g., Roseburia, Butyricicoccus) and reducing pathogenic bacteria (e.g., Parabacteroides johnsonii, Corynebacterium urealyticum); and inhibiting inflammation, apoptosis, and renal fibrosis to mitigate kidney injury (Qian and Shen, 2024; Zhang Y. et al., 2023; Zeng et al., 2024). Metabolomics studies suggest ecdysone and estrone may be its potential active components (Cao et al., 2022).

#### 5.1.3 Fangji huangqi decoction

Fangji Huangqi Decoction originates from the *Essential Prescriptions of the Golden Cabinet* (*Jin Gui Yao Lue*). It consists of *Stephania tetrandra* S. Moore (Menispermaceae), *Astragalus mongholicus* Bunge (Fabaceae), *A. macrocephala* Koidz. (Asteraceae), *Glycyrrhiza uralensis* Fisch. ex DC. (Fabaceae). It is a representative formula for tonifying qi and promoting diuresis. Research indicates that this formula can modulate renal uric acid transport by regulating *LRRK1*-related signaling pathways and upregulating OAT1, OAT3, and ABCG2 expression, thereby enhancing uric acid excretion (Wang et al., 2020). Concurrently, this formula reduces renal and serum expression of IL-1β, NF-κB, EGFR, and *LRRK1*, while elevating PDZK1 levels, thereby exerting anti-inflammatory and renal-protective effects.

### 5.2 Botanical drugs

#### 5.2.1 Heat-clearing and dampness-resolving botanical drugs

Heat-clearing and dampness-resolving agents eliminate internal heat pathogens and promote the expulsion of dampness, commonly used for hyperuricemia associated with damp-heat syndromes.

##### 5.2.1.1 Phellodendron bark

Phellodendron bark, the dried bark of *P. amurense*, is a frequently used treatment for hyperuricemia and a key component in Ermiao San–related formulas. Research indicates that *P. amurense* inhibits hepatic XOD activity, reduces serum and hepatic uric acid levels, modulates inflammatory pathways such as PI3K/Akt, TNF, and NF-κB, decreases inflammatory mediators like IL-6, and lowers serum Cr and BUN levels, thereby improving renal pathological damage (Xu et al., 2022).

##### 5.2.1.2 *Polygonum capitatum* Buch.-Ham. ex D. Don (Polygonaceae)

*Polygonum capitatum*, also known as Shimangcao, is a Polygonaceae herb widely used in Guangxi for urinary system disorders. Research indicates that *P. capitatum* inhibits XOD activity, reducing uric acid production; it downregulates renal URAT1 and GLUT9 expression, promoting uric acid excretion and thereby improving hyperuricemia. Concurrently, it reduces inflammatory factors such as IL-6, IL-1β, and TNF-α, alleviating joint inflammation and improving gout symptoms (Zhang C. et al., 2021).

##### 5.2.1.3 *Clerodendranthus spicatus* (Thunb.) C. Y. Wu ex H. W. Li (Lamiaceae)

*Clerodendranthus spicatus* (Lamiaceae), also known as Kidney Tea or Cat’s Whisker Herb, is used medicinally for its stems and leaves. It has a long history of use in treating gout and urinary system disorders. Research indicates that cat’s whisker herb reduces serum and hepatic XOD and PRPS activity, inhibiting uric acid production; downregulates renal URAT1 and GLUT9 expression to decrease reabsorption; upregulates OAT1 and ABCG2 to promote uric acid excretion; simultaneously lowers serum Cr and BUN levels; improves renal tubular and interstitial damage; and alleviates renal inflammation by inhibiting inflammatory pathways (Wang et al., 2024). Furthermore, cat’s whisker corrects disturbances in fatty acid, glycerophospholipid, bile acid, and purine metabolism. It increases beneficial bacteria such as Roseburia and Enterorhabdus while reducing harmful bacteria like Ileibacterium and UBA1819, thereby improving gut ecology and maintaining uric acid metabolic homeostasis (Chen et al., 2023; Zhu et al., 2023).

#### 5.2.2 Diuretic and dampness-draining botanical drugs

Diuretic and dampness-draining botanical drugs promote urination to expel excess dampness and turbidity from the body, alleviating pathological manifestations associated with “damp stagnation.”

##### 5.2.2.1 Plantago seed

Plantago seed is the dried mature seed of *Plantago asiatica* L. and *Plantago depressa* Willd. (Plantaginaceae). They have a long history of use in hyperuricemia and chronic kidney disease. Studies indicate that Plantago asiatica seeds can inhibit serum XOD activity, reducing uric acid production; downregulate renal URAT1 and GLUT9 expression, decreasing uric acid reabsorption; activate the PPAR signaling pathway to improve lipid metabolism; and lower levels of inflammatory factors (Liu T. et al., 2024).

##### 5.2.2.2 Mulberry bark

Mulberry bark is the dried root bark of *Morus alba* L. (Moraceae), known for its diuretic and edema-reducing effects. Its urate-lowering and renal protective mechanisms include: inhibiting hepatic XOD activity to reduce uric acid production; downregulating renal URAT1 and GLUT9 expression while upregulating ABCG2 to decrease reabsorption and promote excretion; and simultaneously suppressing renal inflammatory responses. Research has also isolated the Diels–Alder compound alkanol A, which has been identified as a mixed-type XOD inhibitor with high development potential (Wang Y. A. et al., 2025).

##### 5.2.2.3 *Cichorium intybus* L. (Asteraceae)

The aerial parts of the Asteraceae plant *Cichorium glandulosum* Boiss. and Huet and *Cichorium intybus*, commonly used as traditional medicine by the Uyghur ethnic group. Research indicates that *C. intybus* enhances ABCG2 expression by regulating the lncRNA H19/miR-21-3p axis, thereby reducing renal uric acid deposition, improving renal function, and alleviating inflammation. Among its components, 11β,13-dihydrocichoriin may be the key active ingredient (An et al., 2025). Furthermore, *C. intybus* modulates gut microbiota to elevate butyrate levels, activates the PPARγ-ABCG2 pathway to promote intestinal ABCG2 expression and enhance uric acid excretion, and upregulates the tight junction protein Claudin-1 to repair the intestinal barrier, thereby maintaining uric acid metabolic homeostasis (Yang Y. et al., 2025).

##### 5.2.2.4 *Hovenia acerba* Lindl. (Rhamnaceae)

The fruit or seeds with fleshy pedicels from the Rhamnaceae plant *Hovenia acerba*. Research indicates that *H. acerba* inhibits XOD activity to reduce uric acid production; upregulates ABCG2 and OAT1 expression to promote uric acid excretion; downregulates URAT1 and GLUT9 expression to inhibit uric acid reabsorption; and modulates hyperuricemia-related inflammatory genes such as ATF3 and CXCR4. It synergistically improves hyperuricemia through multiple pathways (Wang Y. et al., 2025).

#### 5.2.3 Heat-clearing botanical drugs

Heat-clearing botanical drugs directly eliminate pathogenic heat or heat toxins from the body, thereby alleviating “heat syndromes.” Modern medical manifestations often correlate with inflammatory responses.

##### 5.2.3.1 *Paeonia lactiflora* Pall. (Paeoniaceae)

Chishao refers to the dried root of *Paeonia lactiflora* Pall. (Paeoniaceae). Formula data mining indicates that peony-based botanical drugs are frequently used in treating hyperuricemia. Animal and cellular studies confirm that paeonia lactiflora significantly reduces serum uric acid levels: it inhibits XOD activity to decrease uric acid production; downregulates renal URAT1 and GLUT9 while upregulating OAT1 and ABCG2, thereby reducing uric acid reabsorption and promoting excretion. Concurrently, paeonia lactiflora exhibits a favorable safety profile and provides notable protective effects on hepatic and renal tissues (Du et al., 2024).

##### 5.2.3.2 *Thlaspi arvense* L. (Brassicaceae)

It refers to the whole botanical drug or the seeds of *Thlaspi arvense*, employed in Tibetan medicine for the prevention and treatment of hyperuricemia. Research indicates that *T. arvense* inhibits serum XOD activity, thereby reducing uric acid production. It also modulates multiple renal uric acid transport-related genes (SLC22A12, SLC2A9, SLC22A6, ABCG2, SLC22A8, SLC12A1, SLC16A7, SLC4A4), decreasing uric acid reabsorption while increasing secretion and excretion. Additionally, Tribulus terrestris alleviates oxidative stress and inflammatory responses induced by hyperuricemia. Network pharmacology combined with UPLC-Q-TOF-MS analysis indicates its core active components primarily consist of organic acids and flavonoids (Ke et al., 2024).

#### 5.2.4 Tonifying botanical drugs

Tonifying botanical drugs replenish qi, blood, yin, and yang, enhance organ function, and address “deficiency syndrome.”

##### 5.2.4.1 *Gymnadenia conopsea* (L.) R. Br. (Orchidaceae)

The herbal drug hand-shen is derived from the dried tuber of *Gymnadenia conopsea*. Its ethanol extract significantly improves hyperuricemic zebrafish models, with the 95% ethanol extract achieving 84.02% inhibition of XOD. Additionally, the extract markedly reduced reactive oxygen species (ROS) and malondialdehyde (MDA) levels while increasing superoxide dismutase (SOD) activity in hyperuricemic zebrafish, thereby alleviating oxidative stress. Metabolomics studies suggest that its urate-lowering effects may be associated with flavonoids, polyphenols, alkaloids, terpenoids, and phenylpropanoids (Chen T. et al. (2022)).

##### 5.2.4.2 *Astragalus mongholicus* Bunge (Fabaceae)

Huangqi is derived from the dried root of *A. mongholicus*, a representative qi-tonifying Chinese herbal medicine widely used in uric acid-lowering formulations. Research indicates that *A. mongholicus* modulates uric acid transporters by activating the PI3K/Akt signaling pathway, downregulating URAT1 and GLUT9 while upregulating ABCG2, thereby reducing uric acid reabsorption and promoting excretion. Concurrently, it inhibits hepatic XOD activity, decreasing uric acid production and improving hyperuricemia (Zhang M. et al., 2023). Another study indicates that *A. mongholicus*’s uric acid-lowering efficacy is closely linked to the microbiome-metabolite axis. On one hand, it directly modulates metabolic pathways (such as linoleic acid metabolism and glycerophospholipid metabolism). While also increasing the relative abundance of beneficial bacteria (Lactobacillaceae and *Lactobacillus*) and decreasing the relative abundance of pathogenic bacteria (Prevotellaceae, Rikenellaceae, and Bacteroidaceae). This indirectly modulates bile acid metabolism through the microbiota, thereby improving uric acid metabolism disorders (Deng et al., 2023).

#### 5.2.5 Wind-dampness-resolving botanical drugs

Wind-dampness-resolving botanical drugs eliminate wind-dampness and unblock meridians, alleviating symptoms such as joint pain and stiffness associated with “damp-blocking meridians.”

##### 5.2.5.1 *Smilax china* L. (Smilacaceae)

Baqia refers to the rhizome of *Smilax china*. Previous studies have confirmed its effective uric acid-lowering properties in hyperuricemic mouse models. Recent studies in hyperuricemic chicken models revealed that *S. china* inhibits XOD activity to reduce uric acid production; upregulates BCRP and MRP4 expression in kidneys and ileum to enhance uric acid excretion; and decreases the expression of inflammatory factors (IL-6, TNF-α) to mitigate renal injury (Yan et al., 2024).

##### 5.2.5.2 *Ilex cornuta* Lindl. and Paxton (Aquifoliaceae)

Gouguye refers to the dried leaf of *Ilex cornuta*, possessing effects of dispelling wind-dampness and tonifying the liver and kidneys. Research indicates it exerts antihyperuricemic effects through multiple mechanisms: inhibiting hepatic XOD activity to reduce uric acid production; upregulating renal ABCG2, OAT1, and OAT3 while downregulating GLUT9 to decrease uric acid reabsorption and enhance excretion; and simultaneously suppressing NF-κB-mediated inflammatory responses to mitigate renal tubular epithelial cell apoptosis (Zhou et al., 2025).

### 5.3 Active metabolites

#### 5.3.1 Phenols and polyphenols

##### 5.3.1.1 1-(4-Hydroxy-2-methoxyphenyl)-2-(4-hydroxy-3,5-dimethylphenyl)butane-1,2,3-triol

This compound was isolated from *Reynoutria japonica* Houtt. (Polygonaceae). Studies indicate it reduces uric acid production via competitive inhibition of XOD (IC_50_ = 14.35 μg/mL, Ki = 15.29 μg/mL); simultaneously downregulates inflammatory mediators such as TNF-α and IL-6 to mitigate renal injury; and aids uric acid reduction by regulating galactose and purine metabolic pathways (Hu et al., 2023).

##### 5.3.1.2 Punicalagin

Punicalagin (2,3-(S)-hexahydroxydiphenoyl-4,6-(S, S)-gallagyl-D-glucose) is the most abundant tannin in *Punica granatum* L. (Lythraceae). It promotes uric acid excretion by downregulating renal URAT1 and GLUT9 while upregulating ABCG2 and OAT1; simultaneously enhances intestinal excretion by upregulating intestinal ABCG2 and GLUT9; and regulates uric acid homeostasis through multiple pathways by inhibiting renal-intestinal inflammatory pathways, improving renal glucose metabolism disorders, and increasing beneficial bacterial abundance (Han et al., 2025).

##### 5.3.1.3 Saffron petal flavonoid extract (SPFE)

SPFE is derived from the petals of *Crocus sativus* L. (Iridaceae). It inhibits hepatic XOD activity to reduce uric acid production, while upregulating renal/ileal ABCG2 and downregulating URAT1/GLUT9 to enhance uric acid excretion. It also modulates amino acid and lipid metabolic pathways, inhibits the NF-κB inflammatory pathway and oxidative stress, and improves renal pathological damage. Its core components include kaempferol and quercetin derivatives (Chen et al., 2025).

##### 5.3.1.4 Quercetin

Quercetin is a natural flavonoid widely present in various Chinese herbal medicines. It downregulates renal GLUT9 to enhance uric acid excretion; inhibits inflammatory pathways and cytokine production; boosts antioxidant enzyme activity; and suppresses renal cell apoptosis and endoplasmic reticulum stress, thereby improving hyperuricemia and associated renal injury (Liu H. et al., 2025).

##### 5.3.1.5 Glycitein

Glycitein is one of the major reported active components in *Lycium barbarum* L. (Solanaceae). It inhibits XOD activity and stabilizes XDH protein structure, thereby significantly reducing serum uric acid levels (Yang S. et al., 2025).

##### 5.3.1.6 Linarin

Linarin is a key flavonoid component in *Chrysanthemum indicum* L. (Asteraceae). It inhibits XOD and ADA activity to reduce uric acid production; downregulates URAT1, GLUT9, and OAT4 while upregulating ABCG2, OAT1, and OAT3 to enhance uric acid excretion. It also alleviates oxidative stress and improves hepatic, renal, and lipid metabolism (Qian et al., 2025; Jiao et al., 2024).

##### 5.3.1.7 Puerarin

Puerarin is derived from *Pueraria montana* var. *lobata* (Willd.) Maesen and S.M.Almeida ex Sanjappa and Predeep (Fabaceae) and serves as its primary active component. It synergistically reduces uric acid through three mechanisms: inhibiting XOD and ADA activity to decrease uric acid production; upregulating renal and intestinal ABCG2 and renal OAT1 to enhance uric acid excretion; and strengthening intestinal tight junction protein expression, repairing the intestinal barrier, and enriching beneficial bacterial genera to improve gut microbiota (Li et al., 2025).

#### 5.3.2 Glycopeptides

##### 5.3.2.1 Ganoderma lucidum polysaccharide peptide (GLPP)

GLPP is an active extract component of *Ganoderma lucidum* (Curtis) P. Karst. (Polyporaceae). Research indicates that GLPP inhibits ADA activity, reducing the conversion of adenosine to hypoxanthine and thereby decreasing uric acid production. It also downregulates renal GLUT9 and upregulates OAT1, promoting uric acid excretion. Concurrently, it improves renal tissue ATP levels and alleviates tubular damage (Lin et al., 2022).

#### 5.3.3 Saponins

##### 5.3.3.1 Dioscin

Dioscin is derived from the rhizomes of *Dioscorea nipponica* Makino (Dioscoreaceae) and is one of their primary active components. Research indicates dioscin exerts multidimensional synergistic effects in reducing uric acid levels by: reversing abnormalities in the tricarboxylic acid cycle, amino acid, and purine metabolism; corrects lipid imbalances like phospholipids and triglycerides; restores expression of energy metabolism-related genes such as Stx1b; simultaneously mitigates renal injury, downregulates inflammation-associated metabolites, and improves inflammatory states, thereby breaking the vicious cycle of “hyperuricemia–renal injury” (Tan et al., 2021). Its key metabolite, diosgenin, alleviates renal lipid accumulation by upregulating EPHX2 and modulating lipid-metabolism genes, including ACOX1, CPT1α, and PPAR-γ. It simultaneously suppresses expression of renal inflammatory factors (IL-1β, IL-6, TNF-α) and promotes uric acid excretion (Dang et al., 2024).

##### 5.3.3.2 WW-60%-1

WW-60%-1 is a saponin fraction isolated from the roots of *Clematis chinensis* Osbeck (Ranunculaceae), serving as the primary molecular basis for its urate-lowering effects. Research indicates WW-60%-1 modulates urate homeostasis through multi-target synergistic actions: it inhibits hepatic XOD activity to reduce uric acid production; downregulates URAT1 and GLUT9 while upregulating ABCG2, OAT1, and OAT3 to enhance uric acid excretion; suppresses NF-κB/PI3K-Akt pathways to reduce inflammation; alleviates oxidative stress and improves renal-intestinal histopathological damage. Concurrently, NFKB1 has been identified as a pivotal hub gene in its network, regulating multidimensional processes including inflammatory modulation and transport (Liu S. et al., 2025).

#### 5.3.4 Glucosides

##### 5.3.4.1 Total glucosides of herbaceous peony flower (TGPF)

TGPF is derived from the floral parts of the herbaceous peony, *Paeonia lactiflora* Pall. (Paeoniaceae). Studies indicate that TGPF dose-dependently reduces serum uric acid and XOD levels in model rats; downregulates the inflammatory factors TNF-α and MCP-1, along with renal injury markers Cr and BUN; and improves weight loss and pathological damage in the kidney, thymus, and spleen. Its core mechanism involves regulating the renal uric acid transport system: downregulating reabsorption transporters URAT1 and GLUT9 while upregulating excretion transporter OAT1, thereby reducing uric acid reabsorption and promoting excretion (Kang et al., 2020).

#### 5.3.5 Alkaloids and their derivatives

##### 5.3.5.1 Berberine

Berberine is the primary alkaloid in *P. amurense* Rupr. (Rutaceae). Molecular docking studies indicate that berberine exhibits strong affinity for URAT1, downregulating renal URAT1 protein and mRNA expression to inhibit uric acid reabsorption; simultaneously inhibits NLRP3 inflammasome activation, reduces pro-inflammatory factor levels, decreases serum BUN and CRE levels, and improves renal injury (Li et al., 2021).

##### 5.3.5.2 9-Hydroxy-8-oxypalmatine

9-Hydroxy-8-oxypalmatine is an oxidative metabolite of palmatine, the primary alkaloid in *P. amurense*. Studies indicate that it inhibits serum and hepatic XOD and ADA activity, reducing uric acid production; downregulates renal URAT1 and GLUT9 while upregulating OAT1 to regulate uric acid transport; and simultaneously suppresses the NLRP3 inflammasome and downstream inflammatory factors, thereby significantly lowering serum uric acid levels (Wu et al., 2024).

#### 5.3.6 Terpenoids

##### 5.3.6.1 Geniposide derivatives

Geniposide is the primary active component in the fruit of *Gardenia jasminoides* J. Ellis (Rubiaceae) and serves as the precursor for its derivatives (geniposide derivatives). Research indicates that geniposide derivatives inhibit XOD activity to reduce uric acid production; downregulate abnormally activated renal uric acid reabsorption pathways to decrease uric acid accumulation; and suppress inflammatory responses by modulating the TLR4/IκBα/NF-κB signaling axis, thereby improving renal tissue damage and promoting uric acid excretion (Chen J. et al. (2022)).

#### 5.3.7 Polysaccharides

##### 5.3.7.1 Polygonati rhizoma polysaccharide

Polygonati rhizome polysaccharide is a key bioactive component of *Polygonatum sibiricum* Delar. ex Redouté (Asparagaceae). It inhibits XOD and ADA activity and gene expression, thereby reducing uric acid production. Concurrently, it downregulates renal URAT1 expression while upregulating OAT1 and OAT3 expression to enhance uric acid excretion. It also promotes mitochondrial biogenesis, alleviates ROS accumulation and apoptosis in HK-2 cells, thereby exerting renal protective effects (Zhang et al., 2024).

#### 5.3.8 Oligosaccharides

##### 5.3.8.1 Coix seed oligosaccharides (CSO)

CSO is derived from *C. lacryma-jobi L. var. ma-yuen* (Poaceae), obtained through enzymatic hydrolysis and column chromatography purification. Its main component is glucose oligosaccharides with a degree of polymerization of 2–9 (96.5% content). CSO inhibits XOD to reduce uric acid production; downregulates URAT1 and GLUT9 while upregulating ABCG2 and OAT1 to enhance uric acid excretion; suppresses inflammatory responses; improves renal inflammation and lipid metabolism; and increases beneficial gut microbiota abundance. These actions synergistically improve uric acid homeostasis through multiple pathways (Wu et al., 2025).

## 6 Discussion

The onset and progression of hyperuricemia stem from genetic predisposition, metabolic disorders, and the loss of coordinated balance among multiple organs—including the liver, kidneys, and intestines—in the production and excretion of uric acid. In recent years, GWAS studies have provided systematic insights into the genetic basis of serum uric acid levels, identifying key genes such as SLC2A9, ABCG2, SLC22A12, GCKR, and HNF4A, while also revealing population heterogeneity and gene-environment interaction patterns. These findings deepen our understanding of uric acid homeostasis regulation and provide a foundation for subsequent target research and optimization of intervention strategies. Concurrently, TCM research indicates that TCM formulas, botanical drugs, and their active metabolites exert synergistic effects across multiple pathways—including uric acid synthesis, renal and intestinal excretion, energy metabolism, immune inflammation, and gut microbiota, forming a “multi-component, multi-target, multi-pathway” systemic regulatory model that aligns with the complex pathophysiology of hyperuricemia.

Despite advances in both genetics and TCM research, significant limitations persist. First, GWAS primarily identifies statistical associations, with causal genes and molecular functions at many loci remaining unclear, hindering translation of genetic discoveries into therapeutic targets. Insufficient sample sizes, complex gene-environment interaction models, and genetic structural differences across populations also limit the generalizability and interpretability of findings. Second, TCM clinical studies are predominantly small-sample, short-term, and lack multicenter randomized controlled trials, resulting in insufficient evidence for efficacy and safety. In basic research, many botanical drugs and formulas remain at the extract level, with active metabolites, key targets, and synergistic mechanisms yet to be systematically elucidated. Even though tools such as metabolomics and network pharmacology have identified potential pathways, the causal relationships between differential metabolites and uric acid regulation require further validation. Furthermore, research on synergistic mechanisms among multiple active components within formulas, long-term toxicity assessments, and combined applications with existing chemical drugs remains severely limited. These factors constrain the further standardization and in-depth advancement of TCM research.

Although TCM treatment does not rely on precise genetic target matching, its regulated biological processes—such as metabolic pathways, transport networks, and inflammatory responses—overlap with core pathways identified by GWAS. Key genes and associated proteins identified by GWAS may serve as action sites for TCM active metabolites. Consequently, these genetic discoveries provide molecular-level evidence for elucidating the mechanisms underlying TCM efficacy and offer insights for developing multi-target intervention strategies and screening potential drug lead compounds. Conversely, TCM mechanism-of-action research can furnish experimental evidence to support functional interpretation of GWAS results, facilitating the translation of statistical associations into testable biological mechanisms. For instance, its regulation of key transporters such as ABCG2, GLUT9, and URAT1 helps validate the biological significance of relevant genetic loci, thereby enhancing the translation efficiency of genetic discoveries into drug development.

In the future, integrating GWAS and TCM research may provide further support for studies of the HUA mechanism, drug discovery, and intervention optimization. First, technologies like CRISPR/Cas9, spatial omics, and single-cell sequencing should be leveraged to deepen functional studies of GWAS loci, clarifying the tissue-specific roles of key genes within the liver-kidney-gut axis. Second, integrating multi-omics data, including genetic, transcriptional, metabolic, and microbiome information, can construct more comprehensive uric acid regulatory networks. The holistic regulatory properties of TCM offer valuable references for validating the functions of critical nodes within these networks. Third, combining systems pharmacology and natural product chemistry can advance multi-target, mechanism-based lead compound screening or the development of composite intervention strategies. Fourth, integrating phenotypic characteristics such as lifestyle, metabolic status, and TCM syndrome patterns will deepen understanding of the origins of individual variation and support the prediction of treatment response.

In summary, GWAS establishes the genetic framework for hyperuricemia, while TCM reveals potential mechanisms for multi-pathway intervention in uric acid homeostasis from a systemic regulation perspective. Integrating these findings facilitates a multi-level understanding of disease pathogenesis and provides references for optimizing hyperuricemia intervention strategies. With further advancements in functional genomics and systems pharmacology, this interdisciplinary research direction holds promise for providing new support for mechanism studies and therapeutic exploration.
